# Clinical characteristics and prognosis of anal squamous cell carcinoma: a retrospective audit of 144 patients from 11 cancer hospitals in southern China

**DOI:** 10.1186/s12885-020-07170-z

**Published:** 2020-07-21

**Authors:** Yong Lu, Xiaohao Wang, Peiyang Li, Tao Zhang, Jiaming Zhou, Yufeng Ren, Yi Ding, Haihua Peng, Qichun Wei, Kaiyun You, Jason J. Ong, Christopher K. Fairley, Andrew E. Grulich, Meijin Huang, Yuanhong Gao, Huachun Zou

**Affiliations:** 1 School of Public Health, Sun Yat-sen University, Guangzhou, 510080 China; 2 Sun Yat-sen University Cancer Center; State Key Laboratory of Oncology in South China, Collaborative Innovation Center for Cancer Medicine, Guangzhou, 510080 China; 3 Cancer Center, Union Hospital, Tongji Medical College, Huazhong University of Science and Technology, Wuhan, 430022 China; 4 The Sixth Affiliated Hospital of Sun Yat-sen University, Guangzhou, 510655 China; 5 The First Affiliated Hospital of Sun Yat-sen University, Guangzhou, 510080 China; 6 Department of Radiation Oncology, Nanfang Hospital, Southern Medical University, Guangzhou, 510515 China; 7 Department of Radiation Oncology, Affiliated Cancer Hospital & Institute of Guangzhou Medical University, Guangzhou, 510075 China; 8 Department of Radiation Oncology, The Second Affiliated Hospital, Zhejiang University School of Medicine, Hangzhou, 310009 China; 9 Ministry of Education Key Laboratory of Cancer Prevention and Intervention, Zhejiang University, Hangzhou, 310058 China; 10 Department of Radiation Oncology, Sun Yat-sen Memorial Hospital, Guangzhou, 510120 China; 11 Central Clinical School, Monash University, Melbourne, Victoria 3800 Australia; 12 London School of Hygiene and Tropical Medicine, London, WC1E 7HT UK; 13 Melbourne Sexual Health Centre, Alfred Health, Carlton, Victoria 3053 Australia; 14 Kirby Institute, University of New South Wales, Sydney, 2052 Australia; 15 School of Public Health (Shenzhen), Sun Yat-sen University, Shenzhen, 518107 China

**Keywords:** Anal cancer, Squamous cell carcinoma, Treatment, Epidemiology, China

## Abstract

**Background:**

The incidence of anal squamous cell carcinoma (SCC) has been steadily growing globally in the past decade. Clinical data on anal SCC from China are rare. We conducted this study to describe the clinical and epidemiological characteristics of anal SCC in China and explore prognostic factors of outcomes among patients with anal SCC.

**Methods:**

We audited demographic characteristics, relevant symptoms, risk factors, treatment modalities and outcomes for patients diagnosed with anal SCC at 11 medical institutions in China between January 2007 and July 2018.

**Results:**

A total of 144 patients (109 females) were diagnosed with SCC during this period. Median age at initial diagnosis was 52.0 (interquartile range: 46.0–61.8) years. The most common symptoms were bleeding (*n* = 93, 64.6%), noticing a lump (*n* = 49, 34.0%), and pain (*n* = 47, 32.6%). The proportion of patients at the American Joint Committee on Cancer (AJCC) stages I-IV were 10 (6.9%), 22 (15.3%), 61 (42.4%) and 8 (5.6%), respectively, and AJCC stages in 43 (29.9%) patients were unknown. Thirty-six patients (25.0%) underwent abdominoperineal resection initially. Univariable analysis showed that T stage predicted recurrence-free survival (RFS) (Hazard ratio [HR] = 3.03, 95% Confidence interval [CI]: 1.10–8.37, *p* = 0.032), and age group (HR = 2.90, 95% CI: 1.12–7.49, *p* = 0.028), AJCC stage (HR = 4.56, 95% CI: 1.02–20.35, *p* = 0.046), and N stage (HR = 3.05, 95% CI: 1.07–8.74, *p* = 0.038) predicted overall survival (OS).

**Conclusions:**

T stage was identified as prognostic factor of RFS, and age, AJCC stage, and N stage were identified as prognostic factors of OS. Improving symptom awareness and earlier presentation among patients potentially at risk for anal SCC should be encouraged. Familiarity with the standard treatment among health care providers in China should be further improved.

## Background

Anal cancer is a malignancy accounting for 1–2% of digestive tract tumours and 2–4% of colorectal and anal tumours [1–3]. In the general population, anal cancer is rare with an overall incidence rate between 1 and 2/100,000 person-years [4]. It arises from the squamous epithelium of the anal canal and/or perianal skin. Anal cancer can be divided histologically into different subtypes: squamous cell carcinomas (SCC), adenocarcinoma, adeno-squamous carcinoma and melanoma [5, 6]. In Western countries, SCC is much more common than adenocarcinoma. The incidence rate of SCC is steadily increasing throughout the world including in the United States, UK and Australia [7–11]. The main etiological agent for anal SCC is high-risk human papillomavirus (HPV) and hence anal receptive intercourse. HPV-related vulvar or cervical cancer/dysplasia, Human Immunodeficiency Virus (HIV), history of transplantation/chronic immunosuppression all increase its risk [9]. Previous studies also mentioned that the use of tobacco significantly increased the risk of anal SCC [12, 13]. Men who have sex with men (MSM) living with HIV have the highest risk of anal SCC, with an incidence rate of 78/100,000 person-years [14].

Before mid-1980s, the standard treatment for SCC was abdominoperineal resection (APR), however because of several disadvantages, such as permanent stoma, this method as first line therapy was abandoned [3]. In 1974, Nigro introduced combined chemoradiotherapy (CRT) for the treatment of anal SCC [15], which was accepted as a standard treatment after several clinical trials proved its advantages, such as higher local control rates and better organ preservation [16–18]. Surgery is often used for salvage treatment in patients whose local lesions do not respond to treatment and can also be used as primary treatment option when tumours arise from anal margin and be diagnosed at stage I [3].

One of the key factors that influence the outcomes of anal SCC is the stage at diagnosis [19, 20]. A study in the United States indicated that the 5-year survival in patients with a tumour size of ≤2 cm was 85%, but in patients with a tumour size of > 5 cm it was only 45% [21]. The findings from studies in France, Norway and Australia were similar [22, 23]. A study in Norway mentioned that male gender and advanced T-stage could increase the risk of recurrence and death of anal SCC patients [24]. A study in the United States revealed that advanced T stage and immune marker e.g. tumour indoleamine 2,3 dioxygenase 1 (IDO 1) could be used as predictor of recurrence [25].

It is not clear whether the findings from studies carried out in Europe or North America are generalizable to China where literature on anal SCC is scarce. We conducted this study to understand the clinical and epidemiological characteristics of anal SCC and prognostic factors of outcomes in patients with anal SCC in China.

## Methods

We audited anal SCC patients recorded in 11 medical institutions in China between January 2007 and July 2018. (Affiliated Cancer Hospital & institute of Guangzhou Medical University, Guangzhou Panyu Central Hospital, The First Affiliated Hospital of Sun Yat-sen University, Sun Yat-sen Memorial Hospital, The Third Affiliated Hospital of Sun Yat-sen University, The Sixth Affiliated Hospital of Sun Yat-sen University (Guangdong Gastrointestinal Hospital), Sun Yat-sen University Cancer Center, The Second Affiliated Hospital of Zhejiang University, Union Hospital, Tongji Medical College, Huazhong University of Science and Technology, Nanfang Hospital, Southern Medical University, Guangdong General Hospital). Patients with anal SCC were identified using the International Classification of Disease (ICD)-10 codes of anal SCC (C 21.0–21.8) [26]. Adenocarcinomas were excluded. In order to understand the patients’ outcomes more clearly, we conducted a follow-up audit in May 2020 to all patients.

For all identified patients, medical records were reviewed by author PY Li for demographic information (e.g. age of diagnosis, gender, marital status), relevant symptoms (e.g. bleeding, pain, tenesmus, noticing a lump, perianal itch, altered bowel habit or obstruction, etc.), risk factors (e.g. smoking behavior, history of anal sex, history of cancer, HIV status), information of the tumour (e.g. location, histology, tumour size, TNM stage, American Joint Committee on Cancer [AJCC] stage), treatment received (e.g. chemotherapy, radiotherapy, CRT, surgery) and outcomes. AJCC stage was determined according to the American Joint Committee on Cancer, 7th edition [27].

Data obtained from the medical records were summarized using descriptive statistical analysis. Frequencies and percentages were used to describe categorical variables and median and interquartile range (IQR) were used to describe continuous variables. Chi-square test was used to compare proportion of categorical variables. For analysis, age was divided into two groups: ≤50 years and >50 years. Tumour size was divided into two groups: ≤20 mm and >20 mm. Recurrence-free survival (RFS) was defined as the interval between diagnosis and recurrence (local or distant). Overall survival (OS) was defined as the interval between diagnosis and death from any cause or last follow-up [6, 28, 29]. At the last follow-up, patients who did not present an event of interest were censored. Kaplan-Meier analyses were used to evaluate RFS and OS. Cox proportional hazards regression analysis was performed to identify significant prognostic factors of OS and RFS. All statistical analyses were done using SPSS 20.0. A *p* value less than 0.05 was considered to be statistically significant.

This study was approved by the Ethics Committee of the University of New South Wales (IRB ID: HC180393) and the Ethics Committee of the School of Public Health, Sun Yat-sen University (IRB ID: 2018–026). Because the study was retrospective and the data was de-identified, the informed consent requirement was waived.

## Results

A total of 144 patients were identified with anal SCC diagnosed between January 2007 and July 2018 (Table 1). Most (87.5%) patients were diagnosed after 2010. Among these patients, 109 (75.7%) were female. The median age at initial diagnosis was 52.0 (IQR: 46.0–61.8) years (Table 2). 91.4 and 95.4% of male and female patients were married (*p* = 0.637). When grouped by age at initial diagnosis, 41.7% of patients (*n* = 60) were less than or equal to 50 years, and 58.3% of patients (*n* = 84) were older than 50 years.

**Table 1:** Number of patients from participating medical institutions

Medical institution	City	Number of patients
Sun Yat-sen University Cancer Center	Guangzhou	46
The Sixth Affiliated Hospital of Sun Yat-sen University (Guangdong Gastrointestinal Hospital)	Guangzhou	28
Union Hospital, Tongji Medical College, Huazhong University of Science and Technology	Wuhan	19
The First Affiliated Hospital of Sun Yat-sen University	Guangzhou	14
Nanfang Hospital, Southern Medical University	Guangzhou	11
Affiliated Cancer Hospital & institute of Guangzhou Medical University	Guangzhou	9
The Second Affiliated Hospital of Zhejiang University	Hangzhou	6
Sun Yat-sen Memorial Hospital	Guangzhou	5
Guangzhou Panyu Central Hospital	Guangzhou	3
Guangdong General Hospital	Guangzhou	2
The Third Affiliated Hospital of Sun Yat-sen University	Guangzhou	1

**Table 2:** Characteristics of patients, tumour and treatment modalities by gender^a^

Variable	All patient (*N* = 144) n (%)	Females (*N* = 109) n (%)	Males (*N* = 35) n (%)
*Part 1 Demographics*			
Age (Median, IQR)	52.0 (46.0–61.8)	51.0 (45.5–58.0)	60.0 (51.0–72.0)
Age (Range)	17–86	27–84	17–86
Marital status			
Married	136 (94.4%)	104 (95.4%)	32 (91.4%)
Single	2 (1.4%)	1 (0.9%)	1 (2.9%)
Divorced	4 (2.8%)	3 (2.8%)	1 (2.9%)
Widowed	1 (0.7%)	0 (0%)	1 (2.9%)
Unknown	1 (0.7%)	1 (0.9%)	0 (0%)
*Part 2 History of other diseases and symptoms*			
History of smoking			
Yes	17 (11.8%)	3 (2.8%)	14 (40.0%)
No	113 (78.5%)	96 (88.1%)	17 (48.6%)
Unknown	14 (9.7%)	10 (9.2%)	4 (11.4%)
History of cervical cancer			
Yes	–	2 (1.8%)	–
No	–	89 (81.7%)	–
Unknown	–	18 (16.5%)	–
History of vulvar cancer			
Yes	–	2 (1.8%)	–
No	–	89 (81.7%)	–
Unknown	–	18 (16.5%)	–
History of receptive anal intercourse or homosexual behavior			
Yes	0 (0%)	0 (0%)	0 (0%)
No	113 (78.5%)	88 (80.7%)	25 (71.4%)
Unknown	31 (21.5%)	21 (19.3%)	10 (28.6%)
HIV status			
Positive	1 (0.7%)	0 (0%)	1 (2.9%)
Negative	120 (83.3%)	91 (83.5%)	29 (82.9%)
Unknown	23 (16.0%)	18 (16.5%)	5 (14.3%)
Altered bowel habit			
Yes	32 (22.2%)	24 (22.0%)	8 (22.9%)
No	112 (77.8%)	85 (78.0%)	27 (77.1%)
Bleeding			
Yes	93 (64.6%)	75 (68.8%)	18 (51.4%)
No	51 (35.4%)	34 (31.2%)	17 (48.6%)
Pain			
Yes	47 (32.6%)	32 (29.4%)	15 (42.9%)
No	97 (67.4%)	77 (70.6%)	20 (57.1%)
Noticing a lump			
Yes	49 (34.0%)	35 (32.1%)	14 (40.0%)
No	95 (66.0%)	74 (67.9%)	21 (60.0%)
Perianal itch			
Yes	22 (15.3%)	17 (15.6%)	5 (14.3%)
No	122 (84.7%)	92 (84.4%)	30 (85.7%)
Tenesmus			
Yes	20 (13.9%)	14 (12.8%)	6 (17.1%)
No	124 (86.1%)	95 (87.2%)	29 (82.9%)
*Part 3 Characteristics of tumour*			
Tumor site			
Anal margin	14 (9.7%)	8 (7.3%)	6 (17.1%)
Anal canal	119 (82.6%)	95 (87.2%)	24 (68.6%)
Both	5 (3.5%)	3 (2.8%)	2 (5.7%)
Unknown	6 (4.2%)	3 (2.8%)	3 (8.6%)
Tumor size			
≤ 20 mm	35 (24.3%)	30 (27.5%)	5 (14.3%)
> 20 mm	72 (50.0%)	55 (50.5%)	17 (48.6%)
Unknown	37 (25.7%)	24 (22.0%)	13 (37.1%)
T stage			
T1	13 (9.0%)	12 (11.0%)	1 (2.9%)
T2	40 (27.8%)	29 (26.6%)	11 (31.4%)
T3	28 (19.4%)	24 (22.0%)	4 (11.4%)
T4	36 (25.0%)	27 (24.8%)	9 (25.7%)
Unknown	27 (18.8%)	17 (15.6%)	10 (28.6%)
N stage			
N0	52 (36.1%)	42 (38.5%)	10 (28.6%)
N1	33 (22.9%)	25 (22.9%)	8 (22.9%)
N2	22 (15.3%)	16 (14.7%)	6 (17.1%)
N3	8 (5.6%)	7 (6.4%)	1 (2.9%)
Unknown	29 (20.1%)	19 (17.4%)	10 (28.6%)
M stage			
M0	110 (76.4%)	87 (79.8%)	23 (65.7%)
M1	7 (4.9)	5 (4.6%)	2 (5.7%)
Unknown	27 (18.8)	17 (15.6%)	10 (28.6%)
AJCC stage			
I	10 (6.9%)	9 (8.3%)	1 (2.9%)
II	22 (15.3%)	17 (15.6%)	5 (14.3%)
III	61 (42.4%)	48 (44.0%)	13 (37.1%)
IV	8 (5.6%)	6 (5.5%)	2 (5.7%)
Unknown	43 (29.9%)	29 (26.6%)	14 (40.0%)
*Part 4 SCC treatment*			
Chemotherapy			
Yes	107 (74.3%)	82 (75.2%)	25 (71.4%)
No	30 (20.8%)	21 (19.3%)	9 (25.7%)
Unknown	7 (4.9%)	6 (5.5%)	1 (2.9%)
Radiotherapy			
Yes	101 (70.1%)	78 (71.6%)	23 (65.7%)
No	36 (25.0%)	25 (22.9%)	11 (31.4%)
Unknown	7 (4.9%)	6 (5.5%)	1 (2.9%)
Chemoradiotherapy			
Yes	97 (67.4%)	76 (69.7%)	21 (60.0%)
No	40 (27.8%)	27 (24.8%)	13 (37.1%)
Unknown	7 (4.9%)	6 (5.5%)	1 (2.9%)
Surgical operation^b^			
Yes	57 (39.6%)	44 (40.4%)	13 (37.1%)
No	80 (55.6%)	59 (54.1%)	21 (60.0%)
Unknown	7 (4.9%)	6 (5.5%)	1 (2.9%)

^a^: *IQR* interquartile range; *AJCC* American Joint Committee on Cancer; *SCC* squamous cell carcinomas; *CRT* Chemoradiotherapy

^b^: Surgical operation did not include surgical biopsy for diagnosis

Only one (0.7%) patient (male, 45 years old) was recorded as HIV positive. Four (2.8%) patients had a history of other cancers (two cervical cancers and two vulvar cancers). Two (1.4%) patients were diagnosed with sexually transmitted diseases (one syphilis, one had anal intraepithelial neoplasia (AIN) 3 with syphilis and genital warts) while being diagnosed with anal cancer. One (0.7%) patient had a history of genital warts. Among 113 (78.5%) patients with a record of their sexual behaviors, no one reported a history of receptive anal intercourse or homosexual behavior. Seventeen (11.8%) patients were smokers (including current smokers and ex-smokers).

Median duration of symptoms until initial diagnosis was 90 days (IQR: 30–180 days). The most common symptoms were bleeding (*n* = 93, 64.6%), noticing a lump (*n* = 49, 34.0%), pain (*n* = 47, 32.6%), altered bowel habit (*n* = 32, 22.2%), perianal itch (*n* = 22, 15.3%), and tenesmus (*n* = 20, 13.9%). Other symptoms (e.g. constipation [*n* = 2], diarrhea [*n* = 4], thinner feces [*n* = 4], feces with mucus [*n* = 4], and inguinal mass [*n* = 3]) also have been observed. One patient who was diagnosed by physical examination reported no symptom.

The median tumour size was 30.0 (IQR: 20.0–40.5) mm. About one in four patients (*n* = 35, 24.3%) had a tumour less than or equal to 20.0 mm. There were 13 (9.0%) patients diagnosed at T 1 stage, 40 (27.8%), 28 (19.4%), and 36 (25.0%) at stages T 2–4, respectively. The frequencies and proportion of patients in N0–3 stages were 52 (36.1%), 33 (22.9%), 22 (15.3%) and 8 (5.6%), respectively. The frequencies and proportion of patients in AJCC stages I-IV were 10 (6.9%), 22 (15.3%), 61 (42.4%) and 8 (5.6%), respectively, and AJCC stages in 43 (29.9%) patients were unknown.

As for diagnosis and treatment modalities, two patients were misdiagnosed with haemorrhoids and underwent haemorrhoidectomy. Chemotherapy was administered to 74.3% (*n* = 107) of patients, and radiotherapy to 70.1% (*n* = 101) of patients. Ninety-seven (67.4%) patients underwent CRT. Thirty-six patients (25.0%) underwent APR initially, of which four, five, eight, and zero patients were in AJCC stage I, II, III, and IV, respectively, and AJCC stages of the remaining patients were unknown. Of the 36 patients who underwent surgery initially, 24 patients had performed only APR, and eight, three, and one patient had performed CRT, chemotherapy, and radiotherapy after APR, respectively. Tumour sites treated with APR were anal canal or anal canal and margin. Fifteen (10.4%) patients were treated with local mass resection. Only two patients underwent local mass resection were diagnosed at stage I, and sites of tumour were anal margin.

In the current study, seven patients were diagnosed at M1 stage. The metastatic sites were liver in three patients, lung in one patient, sigmoid colon, vagina, uterus, bladder, pelvic, and retroperitoneal lymph nodes in one patient, and left supraclavicular, vena cava and para-aortic lymph nodes in one patient. As for treatment, two patients received induction chemotherapy plus concurrent chemoradiotherapy followed by adjuvant chemotherapy, two patients underwent induction chemotherapy plus concurrent chemoradiotherapy, two patients received chemotherapy alone, and one patient did not receive any treatment. The mainstream chemotherapy regimen was docetaxel plus cisplatin (TP), other regiments, such as fluorouracil plus cisplatin (PF), FOLFOX and Capox were also involved. Among the patients diagnosed at M1 stage, the radiation dose varied from 5400 cGy to 6000 cGy, and the frequency of radiotherapy varied 25 to 30 times. Volumetric intensity modulated arc therapy (VMAT) and intensity modulated radiotherapy (IMRT) were the most often adopted technique. Among these patients, five died of cancer, one survived, and one lost to follow-up.

The chemotherapy regimens were not uniform and varied across different medical institutions. Among all the 144 patients, TP, PF and fluorouracil (5-FU) plus mitomycin were the most widely used. FOLFOX, Capox, capecitabine monotherapy, 5-FU monotherapy, and cisplatin monotherapy were also used in some hospitals. Concurrent chemotherapy was applied to more than half of the patients, and the others were treated with induction chemotherapy, adjuvant chemotherapy or both. For radiotherapy, apart from some earlier patients who adopted 3-dimensional conformal radiation therapy (3D-CRT), the rest patients all adopted VMAT or IMRT technique. Radiation dose had a wide range, fluctuating between 3780 cGy and 7000 cGy. The median of radiation dose was 5600 cGy (IQR: 5000–6000 cGy). The frequency of radiotherapy varied from 21 to 35. Every single dose, with maximum at 240 cGy and minimum at 180 cGy, was performed five times per week.

**Fig. 1:**
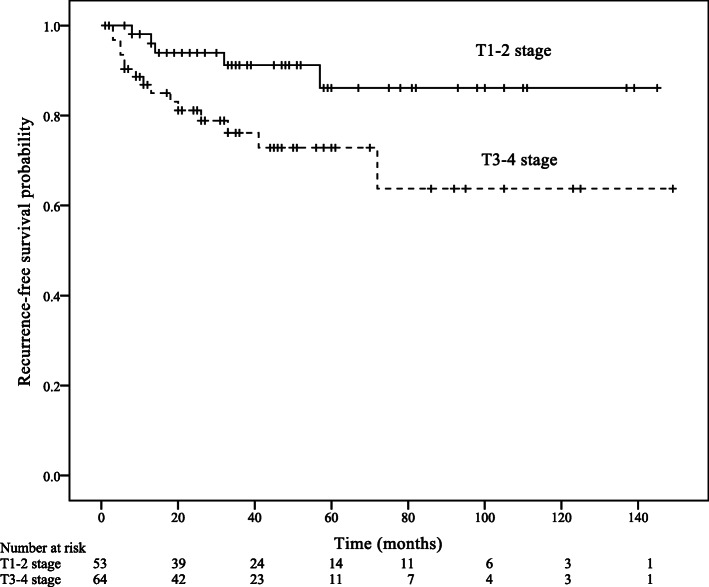
Recurrence-free survival according to T stage

**Fig. 2:**
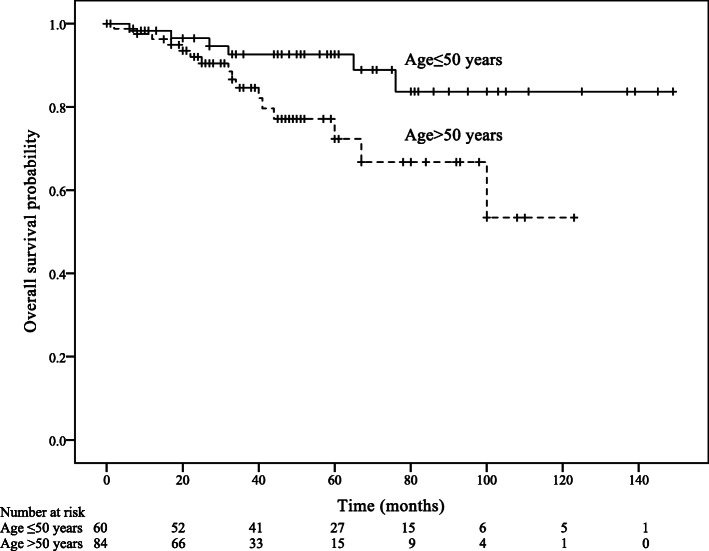
Overall survival according to age group

**Fig. 3:**
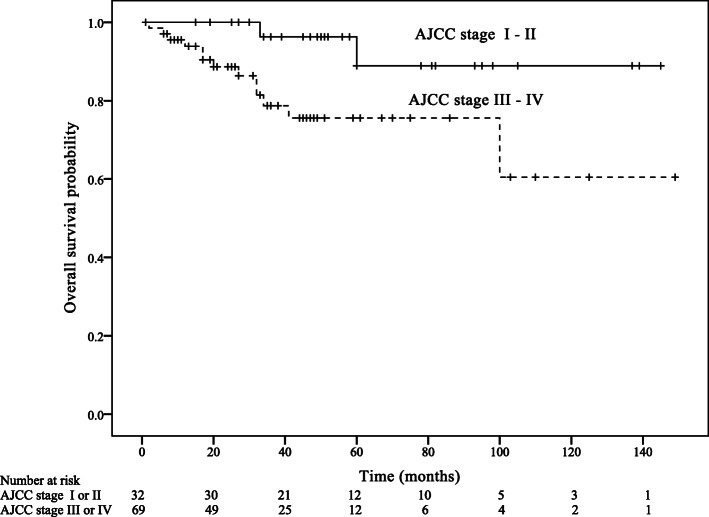
Overall survival according to American Joint Committee on Cancer (AJCC) stage

**Fig. 4:**
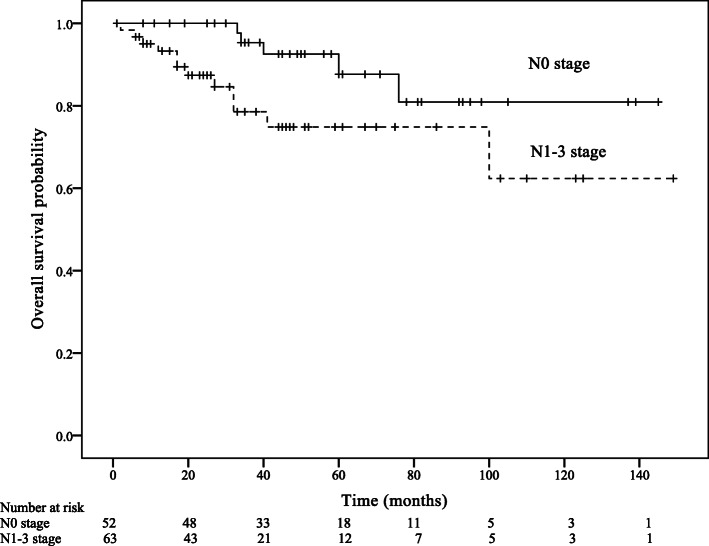
Overall survival according to N stage

Within a median follow-up of 44 months (IQR: 25–67 months), 22 patients died of anal SCC and 25 patients developed a recurrence. Estimated 5-year RFS was 79.4%, and 5-year OS was 82.8%. The univariable analysis of RFS showed that T stage was a significant prognostic factor of RFS (Hazard ratio [HR] = 3.03, 95% Confidence interval [CI]: 1.10–8.37, *p* = 0.032; Table 3; Fig. 1). The univariable analysis of OS showed that age group (HR = 2.90, 95% CI: 1.12–7.49, *p* = 0.028), AJCC stage (HR = 4.56, 95% CI: 1.02–20.35, *p* = 0.046), and N stage (HR = 3.05, 95% CI: 1.07–8.74, *p* = 0.038) predicted OS (Table 4; Figs. 2, 3 and 4).

**Table 3:** Cox univariable analysis for recurrence-free survival (RFS) according to patient, tumour and treatment modalities characteristics^a^

	Univariable	
Variable	HR (95%CI)	*p*
Age (years) (> 50 VS. ≤50)	0.96 (0.43–2.12)	0.918
Gender (Male VS. Female)	1.29 (0.48–3.43)	0.613
AJCC stage (III or IV VS. I or II)	46.17 (0.44–4836.63)	0.106
T stage (T3 or T4 VS. T1 or T2)	3.03 (1.10–8.37)	0.032
N stage (N1-N3 VS. N0)	0.86 (0.35–2.08)	0.730
Tumor size (> 20 mm VS. ≤20 mm)	0.97 (0.33–2.85)	0.960
History of smoking (Yes VS. No)	0.56 (0.13–2.37)	0.431
Chemotherapy (Yes VS. No)	0.62 (0.27–1.41)	0.250
Radiotherapy (Yes VS. No)	0.95 (0.41–2.21)	0.897
CRT (Yes VS. No)	0.75 (0.33–1.68)	0.481

^a^: *AJCC* American Joint Committee on Cancer; *HR* Hazard ratio; *CI* Confidence interval; *CRT* Chemoradiotherapy

**Table 4:** Cox univariable analysis for overall survival (OS) according to patient, tumour and treatment modalities characteristics^a^

	Univariable	
Variable	HR (95%CI)	*p*
Age (years) (> 50 VS. ≤50)	2.90 (1.12–7.49)	0.028
Gender (Male VS. Female)	0.83 (0.33–2.13)	0.702
AJCC stage (III or IV VS. I or II)	4.56 (1.02–20.35)	0.046
T stage (T3 or T4 VS. T1 or T2)	2.33 (0.82–6.63)	0.113
N stage (N1-N3 VS. N0)	3.05 (1.07–8.74)	0.038
Tumor size (> 20 mm VS. ≤20 mm)	0.63 (0.24–1.65)	0.343
History of smoking (Yes VS. No)	1.13 (0.33–3.83)	0.848
Chemotherapy (Yes VS. No)	1.26 (0.46–3.46)	0.652
Radiotherapy (Yes VS. No)	0.81 (0.33–1.96)	0.634
Chemoradiotherapy (Yes VS. No)	0.74 (0.31–1.76)	0.488

^a^: *AJCC* American Joint Committee on Cancer; *HR* Hazard ratio; *CI* Confidence interval; *CRT* Chemoradiotherapy

## Discussion

Our study found that patients with anal SCC were generally diagnosed at late stages. The most common symptoms in patients with anal SCC were bleeding, noticing a lump, and pain. Some patients did not receive standard treatment. T stage was a significant prognostic factor of RFS, and age, AJCC stage, and N stage were significant prognostic factors of OS.

Anal SCC is an uncommon malignancy [7]. Although the incidence rate has been increasing in recent years, it is still difficult to describe the epidemiological characteristics of anal SCC due to its rarity. To our knowledge, our study is by far the largest study in China to describe the clinical and epidemiological characteristics and explore the prognostic factors of outcomes of anal SCC patients. Another study included only 21 anal SCC patients with much more anal adenocarcinomas patients [6].

The smoking rate of different genders in the current study was consistent with the smoking rate among entire Chinese adults. In the current study, 130 patients had the records of history of smoking. Among these 130 patients, approximate 13.1% of patients (45.2% of males and 3.0% of females) had a history of smoking. The smoking rate among males was much higher than that among females. In 2010, Chinese Center for Disease Control and Prevention carried out the Global Adult Tobacco Survey (GATS) in China and reported that smoking rate was 28.1% (52.9% of males and 2.4% of females) among Chinese adults [30]. The China Adult Tobacco Survey (CATS) in 2015 found the smoking rate was 27.7% among Chinese adults (52.1% of males and 2.7% of females), which did not change significantly from the results reported in 2010 [31]. In the current study, 75.7% included patients were female, which resulted in only 13.1% patients had a history of smoking.

Among 144 SCC patients, only a small proportion were diagnosed at an early stage (10 [6.9%] at AJCC stage I and 13 [9.0%] at T1 stage). This was similar to a previous study of anal cancer (129 [71.7%] patients were adenocarcinoma) in China that indicated only 7 of 126 (5.6%) patients were diagnosed at stage I [6]. However, in the United States from 2003 to 2013, more than 30% patients with anal SCC were diagnosed before stage II [32]. The proportion of early diagnosis was significantly higher than that of China. The symptoms reported in our study were similar to those reported in previous studies [6, 23], such as bleeding, pain, noticing a lump, perianal itch, tenesmus, and altered bowel habit. The median duration of symptoms was 90 days and in 39% of patients, symptoms lasted longer than or equal to 180 days. This was presumably the reason why the median tumour size was as large as 30 mm at diagnosis (and the tumour of 34 (23.6%) patients were visible at diagnosis). The long duration of symptoms until initial diagnosis and the large tumour size at diagnosis both indicated that patients were diagnosed too late. Together, these findings suggest that anal SCC could be detected earlier if individuals presented earlier to health care providers [6, 23]. Further studies to improve the rate of early diagnosis of anal SCC should be conducted. Some measures such as high resolution anoscopy (HRA), anal Papanicolau (Pap) smears, and regular digital anorectal examination (DARE) should be implemented in high-risk populations to improve early diagnosis rate of anal SCC [33–38]. HRA is identified as the gold standard for anal cancer screening [34–36]. Anal Pap smears may increase the probability for early diagnosis of anal lesions [37, 38]. An annual DARE could help improve the diagnosis of anal abnormalities, and in high risk population for anal SCC, such as MSM living with HIV, routine implementation of DARE has proven to be cost-effective [33, 39].

In our study we found that about two-thirds of patients received the standard treatment for anal SCC, which was significantly higher than that reported in a previous study carried out in 2011 by Peng et.al where only 9.5% (2/21) received CRT. Peng et al. reported that the reason so few received CRT was that doctors were not familiar with this method as a standard treatment of anal SCC [6]. However, the results of our study also showed issues including nonstandard treatment and misdiagnosis still existed. This pointed to the necessity of education to raise awareness of this condition among both patients and their health care providers, and the importance of early diagnosis and treatment.

Clinical practice guidelines suggest that patients who received the standard CRT could achieve a response rate of 80–90%. The remaining 15% of patients whose regional lesion do not respond to CRT can receive APR as salvage treatment [3]. Among all 144 patients in our study, 36 (25.0%) patients underwent APR initially. Fifteen (10.4%) patients were treated with local mass resection. Only two of them met the conditions for local mass resection. Awareness of anal SCC symptoms and treatment modalities should be further improved among health care providers.

Anal SCC is strongly associated with HPV infection. Recent study regarding cancer burden attributable to HPV infection used 100% as the population-attributable fraction of HPV in anal SCC, which meant that the authors thought nearly all anal SCC were caused by HPV infection [40]. And recent studies regarding the relationship between anal SCC and HPV infection also reported that HPV could be detected in more than 90% patients with anal SCC [41–43]. However, only two patients in our study were determined to be HPV positive. Most patients did not take HPV test, so the HPV infection status were unclear for these patients. Until now, the rate of HPV infection in anal SCC in China, which is of great significance for the exploration of risk factors of anal SCC, is still unknown. HPV test and HPV-related information collection should be conducted among patients with anal SCC in China. Previous studies also mentioned that a prior HPV-related malignancy would increase the risk of second cancer at sites related with HPV, especially among females [44–46]. That is to say, patients with history of cervical, vaginal, vulvar cancers are more likely to develop anal cancer. However, only two patients had a history of cervical cancers, and another two patients had a history of vulvar cancer. Due to limitation of current data, we could not explore the relationship between prior HPV-related malignancy and anal cancer, which might be our future research direction.

In 2012, HPV 16/18 were responsible for 87.0% anal cancer globally, and proportion rose to 95.9% for HPV 6/11/16/18/31/33/45/52/58 [47]. The HPV vaccines have certain potential for the prevention of HPV-related anal cancer. Between 2016 and 2018, bivalent HPV vaccine targeting HPV types 16/18 and nonavalent HPV vaccine targeting HPV types 6/11/16/18/31/33/45/52/58 have been approved in mainland China [48]. However, only right-age females can get HPV vaccine. In China in 2015, the age-standardized incidence rate (ASIR) of anal cancer among males was higher than that among females (0.24 vs 0.17 per 100,000 person-years) [49]. Males, especially MSM, do not have routine access to HPV immunization. Males also should be included in HPV vaccination programs for prevention of HPV-related cancers.

Four factors were identified as prognostic factors of outcomes. In our current analysis, we found that advanced T stage at diagnosis was associated with shorter RFS. Previous conducted in Norway reported that advanced T stage significantly increased the risk of recurrence [24]. Ghosn et al. reported that the status of the margins and tumor size were important predictive factors of recurrence [50]. We also found that age, AJCC stage, and N stage were identified as prognostic factors of OS. Patients diagnosed at a later stage had poorer prognosis which was consistent with the findings from other studies [24, 50]. Elderly patients were more likely have reduced OS, which was in line with the previous study conducted in Norway [24].

Previous studies also mentioned that HPV infection and its surrogate (i.e. p16) were strongly associated with the outcome of anal SCC [51–54]. Compared with patients with HPV-positive/ p16-positive anal tumours, patients with HPV−/p16- tumours had significantly worse outcome. HPV-negative/p16-negative was an independent predictor for reduced locoregional control, RFS and OS [52, 53]. P53 expression was inversely correlated with p16 expression, and p53 positive was an independent prognostic factor for reduced relapse-free survival [53]. TP53 mutations occurred more frequently in HPV negative tumours, which not only was used to predict the outcome of anal SCC, but also related to radiation therapy resistance [52]. However, due to the limitation of our data, we could not explore the relationship between these biomarkers and outcome of anal SCC. Information regarding these biomarkers should be collected in future research.

Anal intercourse, a known risk factor for anal SCC, is practiced in a significant proportion of heterosexual couples (6 to 40%) [55] and nearly all MSM couples. However, no patient in our study reported a history of receptive anal intercourse or homosexual behaviors. This may be a social desirability bias due to the fact that homosexuality and anal intercourse are discriminated against in China and people tend not to disclose their sexual orientation and detailed sexual behaviors, especially for older people. Clinic data relevant to SCC should include sexual behaviors and health care providers should actively collect this information. A large proportion of patients with SCC were HIV-positive and HIV status is associated with younger age at SCC diagnosis. Read et al. reported that nearly 20% (24/128) of patients with SCC in Australia were HIV-positive. HIV-positive patients with SCC had smaller tumours [23]. However, only one patient was recorded as being HIV positive. In China nearly all HIV-positive patients are treated in designated infectious disease specialist hospitals. It is unlikely to have HIV-positive SCC patients at cancer services. In the future, hospitals treating HIV-positive people should be included when collecting data for SCC patients. Exploring the relationship between anal SCC and HIV status is an interesting topic and may be the direction of our next research. The gender ratio of male versus female in our study was 1:3 while a study in Australia found this ratio was 1:1. This may be because the majority of HIV-positive SCC patients were not available at cancer hospitals and a significant proportion of them were MSM. The proportion of receptive anal intercourse among Chinese females was much lower than that among Australian females [56].

There were several limitations that need to be considered when interpreting the data. Our study was a retrospective audit of clinical records. The rarity of anal SCC and the lack of centralised reporting system for anal SCC restricted our ability to find more records which may lead to compromised representativeness of our sample. There is a lack of essential SCC-relevant variables in the current clinical recording system, including detailed sexual behaviors, HPV testing result, HPV-related biomarkers, history of other HPV-related cancers, and HIV status. The tumor registration system in many medical institutions did not systematically separate anal SCC from adenocarcinoma, leading to underreported SCC patients. Meanwhile, the patients in our study were mainly HIV-negative and female. Because of the restricted population, the results of this study may not be generalized. Our study also had several strengths. Our study was the largest one on anal SCC in mainland China. Another strength was the clinical records of patients were collected from 11 medical institutions in various parts of China, which is more representative compared with records obtained from a single institution.

## Conclusions

Our data illustrated that T stage of anal SCC was a predictive factor of RFS, and age, AJCC stage, and N stage were identified as prognostic factors of OS. Among anal SCC patients, only a small fraction were diagnosed at an early stage. The proportion of patients receiving CRT increased over the past decade. However, the usage of CRT still needs to be improved. Further research about identifying other predictive factors of outcomes e.g. biomarkers, improving the rate of early diagnosis and improving the usage of CRT should be conducted. More efforts are needed to collect necessary information regarding HPV infection, biomarkers of HPV infection, history of HPV-related cancer, and sexual behavioral from SCC patients.

## Data Availability

The datasets analyzed during the current study are available from the corresponding authors on reasonable request.

## Abbreviations

SCC: Squamous cell carcinoma
ICD: International Classification of Disease
IQR: Interquartile range
AJCC: American Joint Committee on Cancer
RFS: Recurrence-free survival
HR: Hazard ratio
CI: Confidence interval
CRT: Chemoradiotherapy
OS: Overall survival
HPV: Human papillomavirus
HIV: Human Immunodeficiency Virus
MSM: Men who have sex with men
APR: Abdominoperineal resection
AIN: Anal intraepithelial neoplasia
TP: Docetaxel plus cisplatin
PF: Fluorouracil plus cisplatin
VMAT: Volumetric intensity modulated arc therapy
IMRT: Intensity modulated radiotherapy
5-FU: Fluorouracil
3D-CRT: 3-dimensional conformal radiation therapy
GATS: Global Adult Tobacco Survey
CATS: China Adult Tobacco Survey
HRA: High resolution anoscopy
Pap: Papanicolau
DARE: Digital anorectal examination
ASIR: Age-standardized incidence rate
